# A retrospective study on the relationship between 5 modified frailty index (5-mFI) and postoperative complications of gynecological elderly patients undergoing abdominal surgery

**DOI:** 10.1186/s12871-023-02089-x

**Published:** 2023-04-18

**Authors:** Hai-rui Ma, Jiang Liu, Si-xun Li, Xiao Guo, Yun-feng Zhang, Jing-yan Lin

**Affiliations:** 1 Department of Anesthesiology, The Affiliated Hospital of North Sichuan Medical College, Nanchong, Sichuan 637002 China; 2 Department of Pain Management, Nanchong Central Hospital, Nanchong, Sichuan 637003 China

**Keywords:** Modified frailty index, Elderly patients, Gynecological abdominal surgery, Postoperative complications

## Abstract

**Introduction:**

Aim to evaluate the application of 5 modified frailty index (5-mFI) in predicting postoperative complications in elderly gynecological patients undergoing abdominal surgery.

**Methods:**

A total of 294 elderly gynecological patients who were hospitalized in the affiliated Hospital of North Sichuan Medical College and underwent abdominal surgery from November 2019 to May 2022 were collected from the Union Digital Medical Record (UniDMR) Browser of the hospital. According to whether postoperative complications (infection, hypokalemia, hypoproteinemia, poor wound healing and intestinal obstruction) occurred, the patients were divided into complication group (n = 98) and non-complication group (n = 196). Univariate and multivariate logistic regression analysis were used to analyze the risk factors of complications in elderly gynecological patients undergoing abdominal surgery. The receiver operating characteristic (ROC) curve was used to determine the predictive value of the frailty index score in elderly gynecological patients with postoperative complications after abdominal surgery.

**Results:**

Postoperative complications occurred in 98 of 294 elderly gynecological patients undergoing abdominal surgery, accounting for 33.3%, 5-mFI (OR1.63, 95%CI 1.07–2.46,P = 0.022), age (OR1.08,95%CI 1.02–1.15, P = 0.009), operation time (OR 1.01, 95%CI 1.00-1.01). P < 0.001) were independent risk factors for postoperative complications in elderly patients undergoing abdominal surgery, and the area under the curve of postoperative complications in elderly gynecological patients was 0.60. (95%CI: 0.53–0.67, P = 0.005)

**Conclusion:**

Five modified frailty index can effectively predict the occurrence of postoperative complications in elderly gynecological patients.

## Background

Frailty refers to the non-specific state in which the physiological reserve of the elderly continues to decline, which leads to an increase in social vulnerability and a decrease in anti-stress response ability. When stimulated by the outside world, it can cause complications, adverse events, diseases and other problems. Frailty involves progressive pathological and physiological changes in multiple systems, including neuromuscular, metabolic and immune systems [1, 2]. A large population-based study found that 30% of postoperative deaths are attributable to frailty in older patients [3].

China has entered an aging population socially, and it is estimated that by 2050, the number of people aged 65 and over will reach 400 million, entering super aging. Whether in developed or developing countries, the aging of the population is something that most countries must face up to immediately or in the future [4]. The demand of the elderly population for health services, the economy, and the social pension system is also increasing year by year. However, early studies have shown that the health status of the elderly group is lower than average, 70–80% of the elderly aged 60 and above suffer from chronic diseases, and the elderly group suffers from functional decline, metabolism slowed down, higher prevalence, longer duration of illness and high perioperative risk [5].

At present, the American Society of Anesthesiologists (ASA) class is mainly used clinically, although anesthesiologists are familiar with this, there are considerable differences among anesthesiologists when assigning ASA class to specific patients (for example, elderly patients). ASA physical condition class has not considered age as part of the physical condition system, the aging population is increasing, and more and more elderly people need surgery. In recent years, many studies have proposed to use modified frailty index (mFI)to quantify the degree of the frailty of elderly individuals undergoing surgery. Beyond age, mFI focuses on preoperative indicators, which is a multi-dimensional comprehensive health assessment method, which can more comprehensively and systematically understand the health status of elderly individuals, and predict hospital stay and prognosis [6]. According to statistical analysis, the incidence of perioperative complications and mortality of elderly patients is higher [7]. In recent years, many studies have shown that “frailty” in elderly patients is an important factor in the occurrence of perioperative adverse events [8–13]. In the recent literature, it has also been asserted that frailty should be a standard part of a comprehensive preoperative evaluation and is likely to be included in future revisions to the ASA physical condition system [7].

PaolaAceto et al. [14] conducted a prospective cohort study of 105 patients aged ≥ 65 years who underwent major abdominal surgery and found that 11-mFI was an independent predictor of postoperative pulmonary complications. After 2012, the variables required to calculate 11-mFI were removed from the American College of Surgeons(ACS)-National Surgical QualityImprovement Program(NSQIP) version, 11-mFI is further revised to 5-mFI, including a history of diabetes, a history of chronic obstructive pulmonary disease (COPD), a history of congestive heart failure within 30 days before surgery, a complete or partial dependence on functional health during surgery, and the presence of hypertension requiring drug treatment. Later, studies in multi-subfamilies also showed that 5-mFI is an effective predictor of mortality and postoperative complications in the elderly [10, 15–18].

As far as we know, there are few reports of 5-mFI in abdominal surgery in elderly gynecological patients. Therefore, the purpose of this study was to evaluate the relationship between frailty and postoperative outcome in elderly patients undergoing gynecological surgery.

## Methods

### Study design and subjects

This study was a retrospective cohort study, including elderly gynecological patients who were admitted to the affiliated Hospital of North Sichuan Medical College from November 2009 to May 2022. All patients underwent abdominal surgery under general anesthesia. The Medical Research Ethics Committee of the affiliated Hospital of North Sichuan Medical College approved this study (No.ChiCTR2200055984 ). All methods are in line with the relevant guidelines and regulations of this study.

**Fig. 1:**
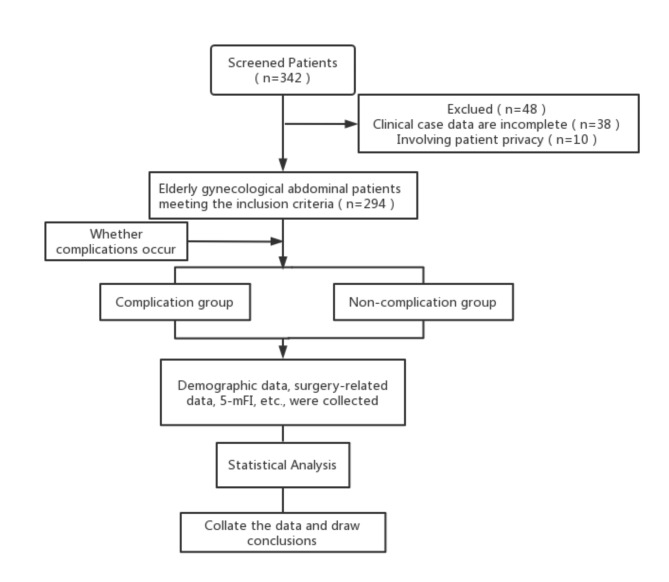
Experimental design flow chart

Patients who met the following criteria were included: age 60 and above; admitted to the affiliated Hospital of North Sichuan Medical College from November 2009 to May 2022 and underwent gynecological abdominal surgery under general anesthesia. We excluded the following patients: the case data were related to patient privacy, the clinical records were incomplete, and the missing item ≥ 1 in 5 items of the modified frailty index. All the data come from the UniDMRBrowser(Version 2012.4, Shanghai Union Network and Information Co.Ltd)of the affiliated Hospital of North Sichuan Medical College. The flow chart is shown in Fig. 1.

### Data collection

Demographic data on patients during hospitalization, including age, body mass index and 5-mFI, were collected retrospectively from medical records. As noted above, 5-mFI is calculated based on the number of comorbid factors:1. history of diabetes, 2. history of chronic obstructive pulmonary disease (COPD), 3. history of congestive heart failure within 30 days before surgery(CHF), 4. complete or partial dependence on functional health during surgery, and 5. hypertension(HTN) requiring medication. The American Cancer Society NSQIP defines functional dependence(FD) as “patients are partially or fully assisted by others in their daily activities 30 days before surgery, including, but not limited to, dressing, bathing, going to the toilet, eating and walking.“The 5-mFI calculations are derived from UniDMR Browser records. History of diabetes, history of COPD, and hypertension requiring medication were obtained from past medical history and admission diagnosis recorded in the UniDMR Browser. The functional status of complete or partial dependence on functional health during surgery was obtained from the Barthel Index Rating Scale in the nursing evaluation sheet in UniDMR Browser. History of congestive heart failure within 30 days before surgery was obtained from the Surgical Thrombosis Risk Assessment Scale (caprini model) in the nursing assessment sheet in UniDMR.We also collected the ASA class, postoperative complications [infection (including wound infection, pulmonary infection, pelvic infection, other infections (patients have abnormal laboratory indicators when reading medical history, infection symptoms such as fever, chills, pain, etc., and effective treatment with antibiotics. However, the exact locations of the infection were not known from the medical records, so it was attributed to another infection.), hypokalemia, hypoproteinemia, etc.], hospital stay, operation time and other data.

### Statistical analysis

Demographic data are classified to two groups as: complication and no complication cohorts.SPSS23.0 statistical software was used to process the data. The measurement data in accordance with the normal distribution are expressed by the mean ± standard deviation('x ± s), the t-test is used for the comparison between groups, and the Mann-Whitney U test is used for those that do not conform to the normal distribution. The count data use case (percentage) indicates that the comparison between groups is made by *X*^*2*^ test. In multivariate analysis, the statistically significant factors in the results of the univariate analysis were selected as independent variables, and the postoperative outcome index as dependent variables, binary logistics regression analysis was used to correct the confounding factors, and the area of receiver operating characteristic (ROC) curve was used to evaluate the predictive value of 5-mFI in postoperative complications. In all analyses, P < 0.05 was considered to be statistically significant.

## Result

From 2019 to 2022, a total of 294 elderly patients underwent gynecological surgery under general anesthesia. The average age of the patients was 66 (IQR:7) years old. In the 5-mFI score, the score of 0 was the most (60.9%), followed by 1 (32.7%) and 2 (5.8%). The incidence of complications was 33.3%. Univariate analysis showed that compared with the non-complication group, the complication group had higher 5-mFI(P = 0.002) and ASA class(P = 0.039), older age(P = 0.011), longer operation time (P < 0.001), and longer postoperative hospital stay (P < 0.001). (Table 1)

**Table 1:** Patient demographics and perioperative risk factors

	Total	Complications	No complications	P value
No.of patients	294	98(33.3%)	196(66.7%)	
Mean age,yrs(M(IQR))	66(7)	67(7)	66(6)	0.011
BMI	23.4(4.0)	24.0(5.0)	23.3(4.0)	0.524
ASA Class				0.039
I	1(0.3%)	0(0.0%)	1(0.5%)	
II	205(69.7%)	60(61.2%)	145(74.0%)	
III	87(29.6%)	38(38.8%)	49(25.0%)	
IV	1(0.3%)	0(0.0%)	1(0.5%)	
Hospital stay, days (M(IQR))	12(7)	15(9)	11(6)	< 0.001
Operation time, mins (M(IQR))	135(95)	162(96)	120(83)	< 0.001
5-mFI				0.002
0	179(60.9%)	46(46.9%)	133(67.9%)	
1	96(32.7%)	45(45.9%)	51(26.0%)	
2	17(5.8%)	6(6.1%)	11(5.6%)	
3	2(0.7%)	1(1.0%)	1(0.5%)	

BMI, body mass index; ASA, American Society of Anesthesiologists.5-mFI, 5 modified frailty index.

Values are presented as median (range interquartile).

^a^ The difference was significant at 0.05 level.

Most of the 5-mFI variables had hypertension, diabetes, history of COPD and partial or full functional dependence accounted for 10.2%,3.1% and 2.0% respectively.

We did not find patients with a history of congestive heart failure within 30 days before the operation. (Table 2)

**Table 2:** Items of 5-mFI

	Number of cases	%
Hypertension + Med	91	31.0
Diabetes	30	10.2
COPD	9	3.1
Functional dependence	6	2.0
Congestive heart failure	0	0

COPD, chronic obstructive pulmonary disease.Med, medicine

The most common complications were infection (20.7%), hypokalemia (11.6%), hypoproteinemia (7.1%), poor incision healing (3.4%), and intestinal obstruction (3.0%) (Table 3).

**Table 3:** Items of Complications

Complications	Number of cases	%
Infection	61	20.7
wound infection	11	3.7
pneumonia	28	9.5
pelvic infection	7	2.4
other infections	15	5.1
Hypokalemia	34	11.6
Hypoproteinemia	21	7.1
Ileus	8	2.7
Poor wound healing	10	3.4

Univariate analysis was performed to determine the significant correlation with postoperative complications in elderly patients undergoing gynecological abdominal surgery. It was found that postoperative complications were significantly correlated with 5-mFI (OR 1.71 95%CI 1.17–2.50; P = 0.022), age (OR 1.07 95%CI 1.01–1.12 P = 0.015), ASA class (OR 1.78 95%CI 1.08–2.95 P = 0.024), and operation time (OR 1.01, 95%CI 1.00-1.01% P < 0.001). There was no significant correlation with BMI. See Table 4 for details. (Table 4)

**Table 4:** Univariateregression analysis of risk factors with complications

	Odds Ratio	95% CI	P value
Age	1.07	1.01–1.12	0.015
Operation time	1.01	1.00-1.01	< 0.001
ASA Class	1.78	1.08–2.95	0.024
BMI	1.03	0.95–1.10	0.496
5-mFI	1.71	1.17–2.50	0.022

OR, odds ratio; CI, confidence interval;ASA, American Society of Anesthesiologists.5-mFI, 5 modified frailty index;BMI, body mass index

^a^ The difference was significant at 0.05 level

Taking the complication (assignment: complication = 1, no complication = 0) as the dependent variable, the factors with statistical significance in the results of the univariate analysis were selected as independent variables, and entered the model when P < 0.05. the results showed that 5-mFI (OR1.63, 95%CI 1.07–2.46; 95%CI P = 0.022), age (OR1.08, 95%CI 1.02–1.15, P = 0.009)and operation time (OR 1.01, 95%CI 1.00-1.01; P < 0.001) were independently associated with complications (Table 5).

**Table 5:** Multivariate regression analysis of risk factors with complications

	Odds Ratio	95% CI	P value
Age	1.08	1.02–1.15	0.009
Operation time	1.01	1.00-1.01	< 0.001
ASA Class	1.10	0.61–1.98	0.753
5-mFI	1.63	1.07–2.46	0.022

OR, odds ratio; CI, confidence interval;ASA, American Society of Anesthesiologists.5-mFI, 5 modified frailty index

^a^ The difference was significant at 0.05 level.

**Fig. 2:**
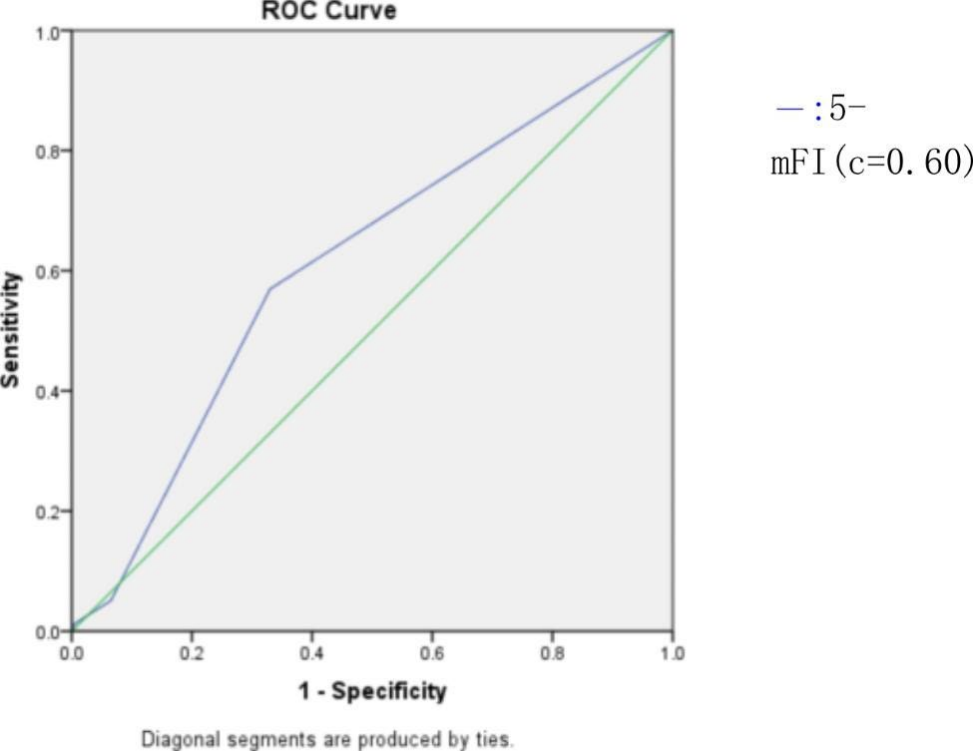
ROC curve of weakness index score for predicting postoperative complications in patients

The area under the curve of postoperative complications in elderly gynecological patients predicted by weakness index score was 0.60 (95%CI:0.53–0.67, P = 0.005). (Fig. 2)

## Discussion

Frailty has become a hot topic in recent years. With the aging of the population in China and even around the world, the frail population is growing, and the number of frail people who need surgical treatment is also increasing year by year. For the identification of frail populations and the risk stratification of postoperative complications, if a simple and accurate tool, such as 5-mFI, is introduced, it can quickly and accurately evaluate surgical patients based on a series of preoperative patient-related complications. It provides a relatively objective means to evaluate the debilitating state of surgical patients and the risk of complications.

In JessicaLuo et al. [19] in 1254 patients who underwent pressure ulcer repair, it was found that 5-mFI was significantly correlated with complications in univariate analysis (OR1.17, CI1.03 ~ 1.33; P = 0.02) and multivariate analysis (OR1.16, CI1.02 ~ 1.34; P = 0.043) compared with other common variables that contributed to risk stratification. Our results show that there is also a significant correlation between the frailty index and adverse outcomes in elderly gynecological patients. The overall complication rate of 294 patients in our cohort was 33.3%. When we controlled the confounding variables, every 1 point increase in the 5-mFI score increased the risk of complications by 1.63 times.

Univariate analysis showed that ASA(OR 1.78 95%CI 1.08–2.95 P = 0.024) grading was associated with complications, but when we controlled confounding variables, we found that there was no significant correlation between ASA(OR 1.10 95%CI 0.61–1.98 P = 0.753)and adverse outcomes. This result might because in clinical practice, anesthesiologists mostly upgrade their ASA Physical Status score according to the extreme circumstances of the patient’s age, which may be independent of the patient’s own systemic disease, or according to the combination of age and systemic disease. Much of this is based on the clinical experience and intuition of the anesthesiologist, which reflects the inherent subjectivity of ASA Physical Status and is difficult to resolve through revision. The propose of frailty, seems to be able to solve this problem well.

We also found age (OR 1.08, 95%CI 1.02–1.15; P = 0.009) and operation time (OR 1.01, 95%CI 1.00–1.0; P < 0.001) were also independently associated with complications. This was consistent with the finding by Osaid Alser et al. [20] that operation time was an independent predictor of infection complications in 4197 patients who underwent ICF repair, and that Barkat Al et al. [21] found an independent predictor of total complications with an age of 30 days in 3237 patients undergoing microsurgical breast reconstruction. However, in 10,550 patients who underwent free flap breast reconstruction, David A. Magno-Pardon [22] et al. found that body weight index greater than 30 kg/m^2^ (OR 1.45; 95%CI,1.31–1.61 ;p < 0.001) was an independent predictor of complications. However, we did not find any association between BMI and postoperative adverse outcomes, which may be due to the fact that there were fewer patients with BMI greater than 30 kg/m^2^ in our observation queue, and this may be due to the lower incidence of obesity among Asian women.

Most of the people we observed had hypertension (31.0%). 10.2% had diabetes, 3.1% had a history of COPD, and 2.0% had partial or full functional dependence. We did not find patients with a history of congestive heart failure within 30 days before the operation. This may be due to the fact that surgeons strictly control surgical indications and carefully screen patients before surgery, or because patients choose delayed surgery or conservative treatment because they had congestive heart failure for nearly a month.

In our study, infection (20.7%) and hypokalemia (11.6%) were the most common complications. When we reviewed the cases, we did not find any patients with a clear diagnosis of “urinary tract infection”. This may be due to the atypical symptoms of urinary tract infection in elderly female patients, and asymptomatic bacterial urine is easy to be missed. In our retrospective analysis, it is also difficult to determine whether the patient’s infection belongs to “urinary tract infection”, so this part of patients suspected of “urinary tract infection” are attributed to other infections.

Postoperative delirium is also a common postoperative complication in elderly patients, which often leads to a series of adverse clinical outcomes and even death. According to the survey, the overall incidence of POD in elderly surgical patients (≥ 65 years old) is 12.0% [23–25]. However, due to its acute onset and fluctuating course of the disease, it is often transient. Patients and their family members have an insufficient understanding of the disease, and fail to respond to the competent physician in time. At the same time, because it is often transient and has not been specially treated in clinic, it may not be recorded in the course of disease. Therefore, after our careful screening of cases, we still did not find any record about postoperative delirium in these elderly patients.

For elderly patients undergoing gynecological surgery under general anesthesia, the 5-mFI predicted the area under the curve of perioperative complications was 0.60 (95%CI:0.53–0.67, P = 0.005), suggesting that 5-mFI can predict perioperative complications in elderly patients undergoing gynecological surgery under general anesthesia. This is consistent with Barkat Ali et al. [21] using the ROC curve to find that mFI seems to be a good predictor of 30-day complications. However, in the study of 5296 patients who received posterior lumbar spondylolisthesis decompression and fusion with Aladine A. Elsamadicy [26]et al., and Sarah Nguyen [27]et al. in 993 patients who underwent transsphenoidal resection of pituitary tumors. They found that 5-mFI and 11-mFI did not seem to predict the occurrence of postoperative adverse events. This may be due to the inconsistency of the criteria for measuring frailty in many literatures, as well as the incomplete understanding of the physiological basis of frailty, and there is no unified consensus on which tool to use. Therefore, this is the limitation of the current use of the frailty scale. The definition of frailty and the use of the frailty scale still need to be further studied. For example, for some patients with hypertension or diabetes, the control of blood pressure and blood sugar during the perioperative period has not been assessed in detail. For some patients with hypertension and diabetes, if it has been well controlled during the perioperative period. This may be different from the occurrence of postoperative adverse outcomes in some patients who fail to receive formal treatment to control blood pressure or blood sugar, which will affect the assessment of the occurrence of adverse events.

To sum up, the frailty scale seems to be able to predict the occurrence of postoperative adverse outcomes. With the aggravation of the aging problem of the population around the world, we should assess the frailty of elderly patients, stratify the surgical risk before the operation, and take certain preventive measures before operation (for example, correcting reversible risk factors, positive medical optimization, optimize preoperative nutritional status). To a certain extent, it can reduce the occurrence of postoperative adverse outcomes, reduce the length of hospital stay, reduce the cost of hospitalization, improve the quality of life of elderly patients and improve the level of medical treatment.

Our next step is to apply the five modified frailty index scales to our patients prospectively. Detailed classification of each frailty index (for example, the investigation of blood pressure control in patients with hypertension and glycosylated hemoglobin in patients with diabetes, etc.), to observe the effectiveness of the frailty index in predicting complications. We will also include the patients’ other preoperative comorbidities (for example, nutritional status, smoking history, alcoholism history, etc.) in the study to observe the extent of their influence on the occurrence of postoperative adverse outcomes. If a correlation is found, we will refine the frailty scale through the study results. More sophisticated frailty scales will be used in the clinic to help doctors better manage patients and optimize their diagnosis and treatment plans.

### Limitation

There are still some limitations in this study. First, this is a retrospective study, so the correlation between variables does not necessarily indicate causality. There is mixed bias, and the information is limited by the accuracy of coding and data input affected by deviation and human error. Incomplete data are eliminated in order to reduce the bias as much as possible. Second, the sample size of this study is small. At present, China is establishing a surgical database and conducting a large-scale epidemiological study on the assessment of the weakness of elderly surgical patients. If we want to do further research, we need a complete database to increase the sample size.

## Conclusion

This study shows that 5-mFI is an effective predictor of postoperative complications in elderly patients undergoing gynecological abdominal surgery. Preoperative assessment of the frailty of elderly gynecological patients can help medical staff to stratify the risk of patients, provide a reasonable basis for clinical diagnosis and treatment, and reduce the incidence of postoperative complications.

## Data Availability

The datasets used and/or analysed during the current study available from the corresponding author on reasonable request.
